# Family management of care for children with chronic conditions in the context of the COVID-19 pandemic

**DOI:** 10.1590/0034-7167-2024-0550

**Published:** 2025-10-03

**Authors:** Nayara Luiza Henriques, Maísa Mara Lopes Macêdo, Melissa Joice de Abreu Felizardo, Elaine Cristina Rodrigues Gesteira, Elysângela Dittz Duarte

**Affiliations:** I Universidade Federal de Minas Gerais. Belo Horizonte, Minas Gerais, Brazil; II Universidade Estadual de Campinas. Campinas, São Paulo, Brazil

**Keywords:** Chronic Disease, Child Care, Family, COVID-19, Qualitative Research., Enfermedad Crónica, Cuidado del Niño, Familia, COVID-19, Investigación Cualitativa.

## Abstract

**Objectives:**

to understand the experience of family management in caring for children with chronic conditions in the context of the COVID-19 pandemic.

**Methods:**

a qualitative study, based on the Family Management Style Framework, carried out with 24 families of children with chronic conditions. Data were collected through semi-structured interviews and subjected to thematic analysis.

**Results:**

before the pandemic, families followed their routines, adapting to care demands. With the pandemic, new challenges in family dynamics were imposed, influencing management behaviors. Coping and resilience were crucial to maintaining focus on the well-being of their children in the context of crisis.

**Final Considerations:**

knowledge about family management of care for children with chronic conditions in the context of the pandemic allowed us to recognize families’ efforts to provide care in adverse situations. New skills and competencies were acquired, but there was an impact on continuity of care.

## RESUMEN

**Objetivos:**

comprender la experiencia de la gestión familiar en el cuidado de niños con enfermedades crónicas en el contexto de la pandemia de COVID-19.

**Métodos:**

estudio cualitativo, basado en el Family Management Style Framework, realizado con 24 familias de niños con enfermedades crónicas. Los datos fueron recolectados mediante entrevistas semiestructuradas y sometidos a análisis temático.

**Resultados:**

antes de la pandemia, las familias seguían sus rutinas, adaptándose a las demandas de cuidado. Con la pandemia se impusieron nuevos desafíos en la dinámica familiar, influyendo en las conductas de gestión. La capacidad de afrontar las situaciones y la resiliencia fueron cruciales para mantener el foco en el bienestar de sus hijos en el contexto de la crisis.

**Consideraciones Finales:**

el conocimiento sobre la gestión familiar del cuidado de niños con enfermedades crónicas en el contexto de la pandemia permitió reconocer los esfuerzos de las familias por brindar cuidados en situaciones adversas. Se adquirieron nuevas habilidades y competencias, pero hubo un impacto en la continuidad de la atención.

## INTRODUCTION

The growth in the number of children living with a chronic condition is a reality identified in recent years^(1)^. This can be attributed to factors such as the increasing specialization of child care and technological advances that allow for more accurate diagnoses and treatments^(2)^. When a child’s health is stable, the home is the main care setting and the family assumes responsibility for this task^(3,4)^.

Throughout life, adaptation and readaptation processes will be experienced by families of children with chronic conditions (CCC)^(5)^. This is because a child’s health condition can vary, as can the family’s finances and support network so that the family’s organization for care will undergo changes over time and according to the context in which CCC and their caregivers are inserted^(3)^. The context, therefore, is understood as the environment in which children and family live^(6)^.

Nurses Kathleen Knafl and Janet Deatrick, authors of the Family Management Style Framework (FMSF)^(7)^, propose the definition of context as a dimension for family care, since it can interfere in the family’s living conditions and in the way it perceives and manages care^(7)^.

A significant change in context was experienced globally with the pandemic due to coronavirus disease 2019 (COVID-19), caused by the SARS-CoV-2 virus^(8)^. Although the literature indicates that children have been less affected by the severe and symptomatic form of COVID-19, CCC have an increased risk of more serious complications and hospitalizations due to infection^(9)^.

In the context of CCC families, the goals of complying with all health regulations and being able to provide continuity of care were considered challenges^(10)^. Additional demands resulting from the pandemic, such as hygiene-related care, mask use and the need for social distancing, have created a greater burden for caregivers and increased stress levels^(11,12)^. In addition to these demands, the family had to deal with the reduction of their support network, family life and care from healthcare professionals^(13)^.

Given this context, it was considered necessary to investigate how families organized themselves to ensure CCC care in the context of the COVID-19 pandemic. To this end, the FMSF was used as a subsidy for this investigation. It is believed that knowing the experience of managing care by families of CCC during the COVID-19 pandemic allows us to identify the potential of the family as a social nucleus of care, its capacity to resist and adapt or not to this type of contextual change, and aspects that may have been weakened during the pandemic, putting family functioning at risk.

Therefore, it is considered that the changes produced in the context of families, brought about by the COVID-19 pandemic, accentuated the family efforts made to meet the care demands of CCC. Based on this assertion, the research question was defined: how was the family management of CCC care during the COVID-19 pandemic?

## OBJECTIVES

To understand the experience of family management in caring for children with chronic conditions in the context of the COVID-19 pandemic in light of the theoretical framework of the Family Management Style Framework.

## METHODS

### Ethical aspects

The project was submitted and approved by the Research Ethics Committee of the proposing institution. In order to ensure the adequacy of this study to the ethical standards of research involving human beings, Resolution 466/2012 of the Brazilian National Health Council (In Portuguese, *Conselho Nacional de Saúde* - CNS) was complied with, as well as the guidelines of Circular Letter 2/2021^(14)^ of the Brazilian National Research Ethics Commission. Informed Consent Form (ICF) was obtained from all individuals involved in the study through oral expression, since the interviews were conducted by telephone due to the social distancing scenario. Thus, the ICF was read and the consent to participate in the study was recorded and archived in secure media as provided for in Resolutions 466/2012 and 580/2018 of the CNS.

### Theoretical-methodological framework

The FMSF^(7)^ was adopted as the theoretical framework for this study, since it allows an analysis of responses of families to the needs experienced due to the existence of a chronic condition in childhood, considering the influence of the context in family life through the following components: definition of the situation (meanings attributed by the family in the experience of illness, identified by the dimensions: child identity, view of illness, management mindset, and parental mutuality); management behaviors (behaviors acquired by family members in different situations exposed by the condition, identified by parental philosophy and management approach); and perceived consequences (current and expected results by the family regarding illness management, identified by family focus and future expectation)^(7)^.

### Study design

This is a qualitative study built based on Consolidated criteria for Reporting Qualitative Research^(15)^.

### Methodological procedures

The research was conducted with 24 CCC families. Initially, a representative of each family was interviewed, 22 mothers, one father and one aunt, considered the main caregivers of children, i.e., those who take the most intense responsibility for child care. In order to contemplate different perspectives of the family, these caregivers were asked to indicate another family member who was also involved in child care. In five families, there was no other caregiver; three did not respond to the request; and 16 caregivers indicated another family member. Of these 16 families, it was possible to interview another family member in nine, six fathers, one mother, one uncle and one grandmother. Therefore, a total of 33 interviews were conducted. It is worth noting that, although the research was not conducted with all family members, the production of information referred to the family as a whole.

Inclusion criteria for participation in the research were being a family member of CCC and being involved in their care, being over 18 years old, able to hear, understand and verbalize the answers to the researcher, and being able to be contacted by telephone. The exclusion criterion was not obtaining a response from the family member after three attempts to contact the researcher. The thematic saturation criterion was adopted to interrupt data collection^(16)^.

### Study setting

The participating families were identified through hospital admission records of children discharged from the Neonatal Intensive Care Unit (NICU) between December 2016 and December 2017, from two reference hospitals for maternal and child health in the capital of Belo Horizonte, Minas Gerais, Brazil. The period was defined to allow the identification of children aged between 2 and 4 years, an age group in which changes in neuropsychomotor development are most noticeable by families^(17)^.

### Data source

Children were identified between October 2019 and May 2020, for a primary study, carried out in the context prior to the COVID-19 pandemic^(18)^. Moreover, 1,115 families were located. Subsequently, telephone contact was made with all these families to apply the Questionnaire for Identification of Children with Chronic Conditions - Revised (QuICCC-R)^(19)^. Of the total number of families, it was not possible to contact 829 because the telephone number was non-existent or no longer belonged to the family member. A total of 286 families were contacted, of which five children had died; 219 did not have chronic conditions according to the QuICCC-R^(19)^; and nine families refused to participate. Thus, 53 CCC families participated in the primary study^(18)^.

For this research, attempts were made to contact the same 53 families participating in the primary study^(17)^. Of these, nine families could not be contacted because the telephone number was not valid; 13 did not respond after three attempted calls; and four did not wish to participate. Thus, data collection was carried out with 27 families; however, three participated in a pilot test and were not part of data analysis, thus leaving 24 families.

### Data collection and organization

Data collection took place between January and May 2021, through semi-structured interviews. Due to COVID-19 infection prevention measures, it was decided to produce the data through telephone contact, which ensured access and reach to families in the context of social isolation. To ensure the effectiveness of the interview, participants were initially contacted by the researcher of the primary study with whom they were already familiar. At the time, she introduced this study and the researchers involved in it, facilitating contact. Recognizing the potential limitations of telephone interviews, such as participant distractions, technical and connection problems, lack of nonverbal communication and weaker connection between researcher and interviewee, strategies were adopted such as scheduling the interview in advance to minimize distractions, maintaining contact with interviewees via text messages on WhatsApp^®^ to strengthen the bond and creating an interview script with questions to facilitate the proper conduct of the interview.

The interview script was prepared according to the FMSF components^(7)^, with questions about daily care of children during the pandemic, focusing on changes in care demands, family dynamics, family mutuality, access to healthcare services, and perspectives on children’s future. The interviews were conducted by one of the authors of this study, after prior telephone contact with participants to schedule a day and time of their preference. The researcher is a nurse, master’s and doctoral student in nursing, with experience in conducting qualitative interviews and, although she worked in child care at one of the hospitals in which participants were identified, she had no prior contact with them.

The interview was conducted individually with each member of the participating family. Care was taken to ensure that the environment was quiet and had good telephone signal coverage. The average length of interviews was 15 minutes and 34 seconds. All interviews were transcribed in full. To validate each transcription, the text was read and the audio was listened to.

### Data analysis

The data were subjected to thematic analysis^(20)^, with a deductive approach guided by the FMSF. The transcribed interview documents were exported to the MAXQDA^®^ software^(21)^ for qualitative data coding, exploration and management.

To construct the thematic analysis, the six stages proposed by Braun and Clarke were used^(20)^. Initially, themes corresponding to the FMSF components and dimensions were listed^(7)^. These themes were applied to the interview transcripts and subsequently we sought to identify aspects that signaled patterns in families’ experience. To validate theme assignment, the first five interviews were coded independently and blindly by two researchers, the first and second authors of this study. At this stage, a Kappa index of 0.95 was obtained, which represents a high level of intercoder agreement^(22)^. Any discrepancies found were discussed with a third researcher to reach consensus. The remaining interviews were coded by the second author of this article.

## RESULTS

### Participant characterization

Twenty-four CCC families participated. It is considered that, since a primary caregiver is a person who takes on the most intense care of children, they can provide information about the family’s perspective. Therefore, the sociodemographic characteristics that will be presented refer to primary caregivers. The majority (n=22) of interviews were answered by mothers, of whom 41% were housewives. Among the primary caregivers (n=24), the predominant age range was 31 to 40 years (n=17). The majority (n=8) had completed high school, followed by higher education. As for ethnicity, four were white, four were black, and 16 were of other ethnicities. The majority (n=21) had some religion and lived with a partner (n=18). Family income was based on the minimum wage (R$1,100.00), with 25% receiving less than this. Seventeen families received some type of financial aid.

The 25 children participating were between 3 and 4 years old, with a mean gestational age of 31.8 weeks, and nine were premature. The majority were male (n=17), and 12 attended school. All received specialized healthcare, with the majority (n=13) requiring multiple specialties. Diagnoses included neurological and/or neuromuscular conditions (n=13), cardiovascular conditions (n=3), respiratory conditions (n=1), gastrointestinal and endocrine conditions (n=1), renal conditions (n=1), bone and/or joint conditions (n=4), and congenital anomalies and genetic defects (n=2). Children with hydrocephalus used ventriculoperitoneal shunt.

### Family Management Style Model

**Figure 1:**
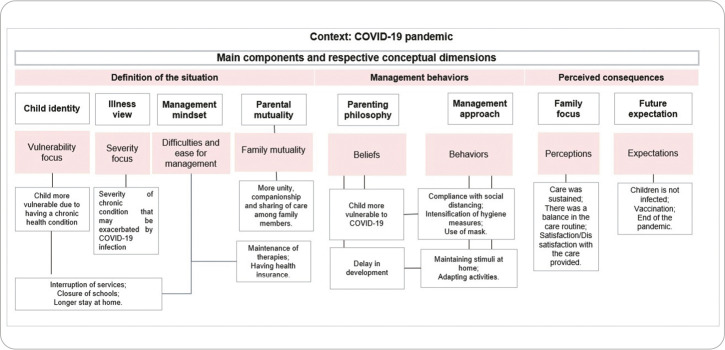
Family Management Style Model for children with chronic conditions in the context of the COVID-19 pandemic, Belo Horizonte, Minas Gerais, Brazil, 2024

*Source: adapted from Knafl, Deatrick and Havill (2012).*

Data analysis allowed us to identify, based on the FMSF components, elements that comprised the experience of childcare provided by families during the COVID-19 pandemic. Figure 1 allows us to visualize aspects of this experience as well as the changes experienced in this new context of care.

### Definition of the situation

This component was designed to identify how families experienced the care of CCC during the COVID-19 pandemic. According to the FMSF, the definition of the situation is related to the meaning that family members attribute to important elements of the situation experienced, which in this study is specifically the care of a CCC in the context of the pandemic. Four dimensions make up this component: child identity; view of illness; management mindset; and family mutuality.

Child identity was expressed through the weaknesses identified by family members considering pre-existing health conditions and their contribution to greater susceptibility to COVID-19. Families’ view of illness reflected a negative prognosis for children, due to the uncertainty of the end of the pandemic, negative information in the media about infected people, lack of knowledge about the consequences of the illness, and uncertainty about vaccination. Families’ management mindset during the pandemic highlighted difficulties resulting from the interruption of services, school closures, and increased stress for children due to spending more time at home. Despite the challenges imposed by the pandemic, family mutuality stood out for the opportunity for unity, companionship, and participation in care among family members. The information is exemplified in Chart 1.

**Chart 1:** Defining the situation of families to manage children with chronic conditions during the COVID-19 pandemic, Belo Horizonte, Minas Gerais, Brazil, 2024

Category: definition of the situation		
**Dimensions**	**Family management experience**	**Examples of statements**
**Child identity:** parents’ views of the child and the extent to which those views focus on illness or normalcy and capabilities or vulnerabilities.	Weaknesses caused by chronic conditions leading to greater susceptibility to COVID-19 infection.	[...] *I’m afraid he’ll catch COVID, and since he has respiratory problems, he’ll have extreme difficulty breathing* [...]. (M43) *A lot of things have changed because we’re scared, you know? She’s very fragile. If she gets on this train, I think it’ll be terrible, you know?* (P22)
**Illness view:** parents’ beliefs about the cause, seriousness, predictability, and course of the illness.	Families believe that existing chronic conditions are serious and that they can be exacerbated if children become infected, which causes anxiety and fear.	*She has lung dysplasia. So, her having these lung lesions makes me worried about COVID, because it is a respiratory virus.* (M5) *If he catches the virus, he could get worse. The biggest concern is related to that, right? They said there won’t be a vaccine for him or anything, that they won’t give the vaccine to children. I thought it was absurd, right? He needs the vaccine.* (M24)
**Management mindset:** parents’ views of the ease or difficulty of carrying out the treatment regimen and their ability to manage effectively.	The facilities acquired throughout the management experience before the pandemic were impacted by the interruption of assistance services, making it difficult to follow the therapeutic regimen.	*As they say, during this pandemic, we had to stop swimming lessons. We didn’t have any appointments at the outpatient clinic at Hospital das Clínicas here at APAE. The doctor’s office also stopped, so care became difficult* [...]. (M50) *It’s been really busy, right? More stressful, because there’s no school, right? So, they stay home and are more anxious.* (M20)
	Need for caregivers to maintain therapies at home.	*Well, it’s being like this, more delicate, because she’s not having physical therapy, right? She’s not having these in-person appointments. So, in this case, she’s having online physical therapy, right? In this case, I’m the one having to do physical therapy.* (M25)
**Family mutuality:** caregivers’ beliefs about the extent to which they have shared or discrepant views of the child, the illness, their parenting philosophy, and their approach to illness management.	Family members had already become more involved in caring for children, becoming more united, but with the pandemic, this experience intensified.	*He also helps me a lot* [referring to her husband]. *He just doesn’t help me with the tube, because, like, he’s very insecure about the tube.* (M25) *Actually, even before the pandemic, we got closer because of C. We dedicated ourselves a lot to her, really a lot.* (P5)

### Family behaviors for managing care for children with chronic conditions during the COVID-19 pandemic

Management behaviors refer to the family’s efforts to care for and adapt to the demands arising from the chronic condition. Two dimensions help to understand this component: caregivers’ philosophy; and management approach.

Caregivers’ philosophy encompasses their beliefs, values, and priorities, directly influencing the management approach. In this study, it was identified that the belief in children’s susceptibility to COVID-19 was a determining factor in intensifying care to prevent infection and maintain their health (management approach), such as avoiding leaving the house, limiting visits, and intensifying hygiene measures. The belief (caregivers’ philosophy) that the lack of professional stimulation and socialization can lead to delays in children’s development led families to incorporate stimulation activities at home (management approach). Information about this component is illustrated in Chart 2.

**Chart 2:** Family behaviors to manage care for children with chronic conditions during the COVID-19 pandemic, Belo Horizonte, Minas Gerais, Brazil, 2024

Component: management behavior		
**Dimensions**	**Family management experience**	**Examples of statements**
**Parenting philosophy:** parents’ goals, priorities, and values that guide the overall approach and specific strategies for illness management.	Belief that children are more vulnerable and susceptible to COVID-19.	*Well, we’ve redoubled our care because she has this part of the gastrostomy. We’re very concerned.* (P2) *So, we’re even more careful, now it’s double. The care I used to take with him was mainly with his food. Now, you know, I have to avoid going out in public, use hand sanitizer, and clean myself as much as possible.* (M17)
	Belief that the lack of stimuli from specialized professionals and the impossibility of socialization can lead to delays in development	*Ah, one way or another, development is affected, right? Because everything has stopped, school has stopped, speech therapy, physiotherapy, so, whether you like it or not, everything is being affected, because you don’t have all the resource.* (M16) *My daughter will be affected in every way. She needs to reinforce her steps, she has occupational therapy, she has physiotherapy, and so far, nothing. She needs it!* (M5)
**Management approach:** development of strategies and routines for managing the child’s condition	Adoption of behaviors to comply with social distancing, intensification of measures to prevent and contain COVID-19.	*We avoid having too many people over because of the C41. We don’t go out, only when we need to.* (M41) *We’re already afraid because of her immunity, which is already low, right? And if I need to go out, I leave her at home.* (M51) *If you have to go out, you need to wear masks, be careful with hand sanitizer, and you can’t let anyone touch it.* (M24)
	Adoption of behaviors to maintain child stimulation activities at home.	*I do physiotherapy with her here at home. We play a lot, I move with her, so she ends up doing physiotherapy.* (M22) *He had done eight sessions. The physiotherapist stopped seeing her, so she asked to continue doing it at home.* (M53)

### Consequences perceived by family members regarding caring for a child with chronic conditions in the context of the COVID-19 pandemic

This component concerns the family’s assessment of the impact of the chronic condition and resulting care on family functioning and future expectation. In this study, this assessment considered changes in family dynamics due to the COVID-19 pandemic. According to the FMSF, the component has two dimensions: family focus; and future expectation.

Concerning family focus, which involves satisfaction with the incorporation of the management of the child’s condition into family life, caregivers reported a balance in their care routine, despite the increased responsibilities due to COVID-19 containment measures. They focused on protecting, preserving the health and promoting the well-being of CCC, maintaining the usual care activities and compensating for those affected by the pandemic, such as stimulating development and rehabilitation. Most caregivers expressed satisfaction with the new routine, attributing it to efforts to ensure quality of care. However, some expressed dissatisfaction, feeling inadequately trained compared to health and education professionals.

Regarding future expectation, all caregivers were asked about the future of children and the family in the context of the pandemic. Only 12 responded, while the others were evasive, possibly due to the uncertainty of the situation. The future expectation expressed included the desire for children not to contract the virus and the hope for the end of the pandemic and the start of vaccination. Information on this component is exemplified in Chart 3.

**Chart 3:** Consequences perceived by family members regarding caring for a child with chronic conditions in the context of the COVID-19 pandemic, Belo Horizonte, Minas Gerais, Brazil, 2024

Component: perceived consequences		
**Dimensions**	**Family management experience**	**Examples of statements**
**Family focus:** parents’ assessment of the balance between illness management and other aspects of family life.	Assessment that child care was sustained through the construction of an adjusted family routine.	*M18 is the pillar here at home. I’m out there working hard and she’s here inside with him, teaching, educating, taking care of him.* (P18) *I’m practically all for her, right? When she’s free, I go and do some laundry, or clean the house.* [...] *my day and my night. I live for her.* (M8)
	Assessment of satisfaction with the care provided.	*We try to provide 100% care. Of course, we can’t do it, but 90%, our best, we can do it.* (M18) *No one can be 100%, but we try to be as observant, attentive and responsible as possible for the situation, taking precautions.* (P2)
	Dissatisfaction with the care provided due to the belief that some care cannot be performed well.	[...] *everything stopped, school stopped, speech therapy, physiotherapy stopped, so, whether you like it or not, it’s affecting everything, right, because you don’t have all the resources. Here at home, I talk, I try, I encourage her a lot, I play, but it’s not the same thing as a professional doing with her, you know, being with her.* (M16)
**Future expectation:** parents’ assessment of the implications of the illness for their child’s and family’s future.	Expectation of non-contamination of the child	*I hope C8 doesn’t get it, because I’m really scared. She has a punctured heart, lung problems, a bunch of little things, you know?* (P8)
	Hope for the arrival of the COVID-19 vaccine.	*I’m rooting for this vaccine, because, wow, to leave this worry behind* [...]. (M3) *Look, now, with the vaccine arriving, right, we’re a little hopeful, right, but I’ll tell you it’s a lot of stress* [...]. (M17)
	Hope for the end of the pandemic.	[...] *hope the pandemic ends soon, everyone gets vaccinated, right, he goes to school!* (A24)

## DISCUSSION

The COVID-19 pandemic has triggered substantial changes in the routine of families caring for CCC, influencing the management of daily care and creating new needs. Understanding how families have incorporated CCC care into their daily family life, as well as the challenges of providing care during a health crisis, has highlighted the need to offer support to these families, based on recognizing how they perceive and assess CCC and the care provided, as well as the resources they use as a source of support and guidance.

This study shows that families who faced the most intense challenges related to caring for CCC during the pandemic were those with lower monthly family income and who had children with complex neurological conditions, as these families were already facing financial difficulties before the pandemic, as they needed to dedicate themselves fully to caring for their children. With the pandemic, they needed to incorporate new care into their routine, in addition to experiencing the loss of their support network, which aggravated the situation of social and financial vulnerability and emotional overload. It is known that families with CCC have a more vulnerable financial condition, resulting, among other things, from the interruption of paid work to dedicate themselves to care, in addition to health expenses. These events can accentuate the reduction in family income, compromising the family’s management and adaptation to adverse situations^(23)^.

As a strategy to mitigate the economic crisis resulting from the pandemic, aiming to guarantee minimum income and food security, the Brazilian government offered emergency aid. This financial increase was intended for families in a situation of economic vulnerability^(24)^. In this study, most families received this aid. Although this government action seems to have contributed to maintaining the economic stability of vulnerable CCC families, this group needs to be the target of effective and ongoing social security strategies, not limited to periods of crisis. It is important to highlight that families who reported greater purchasing power in this research also faced changes in their care routine, since, with the circumstances of the pandemic, it was not possible to maintain normal routines.

Data analysis based on the FMSF revealed the existence of an interconnection between the dimensions of the theoretical framework, which was essential to understanding the changes that occurred in the routine of family care during the pandemic. The findings of this study indicate that the pandemic influenced caregivers to emphasize their children’s weaknesses to the detriment of their potential. By attributing this identity to the child, their belief that they are more susceptible to COVID-19 and its repercussions is also reinforced, in addition to the view that existing chronic conditions can be exacerbated if children are infected. This led families to experience feelings of fear and concern, and to adopt management behaviors aimed at protecting the child against SARS-CoV-2 infection, such as the use of alcohol gel, masks and social distancing, with the future expectation that the child would not become infected.

The beliefs that define the family’s view of children’s condition can be modified depending on the context in which the family is inserted^(7)^. Considering the data collection period, it is believed that the uncertainty regarding the end of the pandemic, the bad news spread by the media, the insecurity regarding the infection and its consequences, and the uncertainty regarding when the vaccine would be a reality may have contributed to the belief in a poor prognosis for CCC. Unlike adults, most infected children had a milder course of the illness. However, most deaths, hospitalizations, and critical consequences reported in children occurred in those with previous comorbidities^(9)^, data that justify greater concern among caregivers for their children, as well as the adoption of precautions to prevent COVID-19.

The interruption of healthcare services and the closure of schools, difficulties identified by caregivers, were linked to the belief in the possibility of harm to children’s development. As a management approach, families made an effort to carry out stimulation activities at home and remained closer together, as evidenced in the family mutuality dimension. Therefore, the results allow us to recognize that caregivers already had knowledge and skills prior to the pandemic, resulting from continuous learning and constant adaptations regarding the needs of their child and care, supporting actions when access to health and education services was lost.

Studies indicate that the COVID-19 pandemic has had significant impacts on child development, with preschool and school-aged children being the most affected. Increased exposure to screens, reduced outdoor play and educational activities, increased food insecurity, and reduced social contact with family and friends have been identified as causing socio-emotional, linguistic, growth and developmental impairments related to mental health^(25,26)^. This context indicates the need for families to be supported in managing children in situations that change their life contexts, reducing the impacts on child development. For example, there is the implementation of actions such as teleconsultations, remote educational support, caregiver training programs and the creation of materials and accessible technologies that act to favor the development of children at home.

Participants in this study also reported that family members remained at home for longer during the COVID-19 pandemic, a fact reported as a difficulty, but which also provided more family life and participation in CCC care. Participants’ perception of “perceived consequences” shows satisfaction with the joint effort in adopting protective measures against COVID-19. For some families, this perception is enhanced by the recognition of family mutuality, which, in addition to sharing care, has accentuated unity and companionship among family members. It is worth considering that family mutuality is the result of an ongoing process of construction within the family that may have been strengthened during the pandemic period, but is not limited to it.

Most of the interviewees had high school or higher education, an aspect that may have contributed to childcare, since there is a positive relationship between years of education and the ability to manage childcare^(23)^. Caregivers’ competency for care was a central component verified in family behavior in the “management approach” dimension. Although the relevance of this competency for harm reduction and the family’s power as a support for CCC care is evident, it deserves attention, especially in the medium and long term, in similar health situations. One aspect to consider is the preparation of caregivers to meet already known needs. Changes in children’s development and health require actions that caregivers may not have experienced before.

Therefore, although interventions carried out at home have played an important role in the rehabilitation of children in an adverse context, this strategy may not be sustainable over time. Caregivers may not have been trained to act appropriately to achieve the desired functional results, justifying the dissatisfaction with the care reported by some of them. Furthermore, even if trained, care for children by a qualified professional is not dispensable, since it is carried out individually, according to the development of each child, and it is important for the family to recognize this aspect.

The interruption or modification of care aimed at the rehabilitation of children has the potential to harm their functional capacity^(27)^. In this study, it is clear that families recognize this impact, justifying their concern with maintaining and ensuring stimulus activities. Resources that facilitate the sharing of care remotely should be used more, such as telehealthcare services and the use of video calling platforms, aiming to prevent the negative effects of future health emergencies that require social distancing.

Studies indicate that the loss of support offered by schools was one of the greatest challenges for CCC families^(28,29)^. In this research, this challenge was crucial for adopting behaviors as a management approach, such as dedicating more time to school activities at home, aiming to minimize the impacts of the lack of education and socialization of children in schools. Factors such as learning difficulties, children of different ages at home and the need to work may have exacerbated the challenges^(26)^.

The impossibility of children socializing with their peers, caused by school closures and social distancing, was another cause for concern for some caregivers. The confinement caused by the pandemic brought limitations to children’s lives, restricting their environment to their homes and interaction with their families. In fact, depriving children of social interaction can lead to losses in their learning and development. Furthermore, in this study, caregivers noticed that the lack of interaction with other children may have been a cause of irritability, fear, and behavioral changes in their children, findings that are consistent with a study of children and adolescents with sickle cell illness^(30)^.

### Study limitations

The study’s limitation is the population selected, which is considered to be not very diverse. Investigations with specific CCC families and with more complex care demands may produce different results than those found in this study.

### Contributions to nursing

This study contributes to nursing because it uses the FMSF as a theoretical model that can guide nursing practices, providing critical reflections on: the dynamics of each family, including their view of the child and their health condition; the efforts needed to establish a routine and meet the demands of CCC; the impact of CCC’s health condition on the family and the future; and the influence of the context in which the family is inserted.

## FINAL CONSIDERATIONS

The pandemic has led to changes in the daily lives of CCC families, creating unique challenges to be faced. The change in routine was necessary, especially due to the incorporation of precautions for the prevention and containment of COVID-19, such as hygiene measures, use of masks and social distancing. In addition, families experienced the interruption of healthcare services and the closure of schools, which were seen as a hardship due to the potential to harm children’s development.

The substantial and rapid change in the way health and education professionals offer and access support has increased the burden on caregivers, who have now dedicated themselves to teaching school activities and rehabilitation at home. However, despite the difficulties experienced, families have made an effort to ensure childcare, becoming more united as they spend more time at home.

It is important to note that, although the family is the protagonist of home care and has the skills and competency to perform this role, this does not mean that it should assume this responsibility exclusively. The loss of support during the pandemic highlighted the capabilities of CCC families, but it also highlighted the importance of professionals as partners in this care. Therefore, in adverse contexts and social distancing, it is necessary to think of strategies that strengthen this partnership and ensure the continuity of care in a structured and accessible manner, using existing knowledge about the needs of children, conditions that favor development, and technology mediation both for care directed at children, such as teleconsultations, and for health education for their caregivers.

Even recognizing the contribution that practices adopted by professionals can make to improving care in situations of changing care contexts, it is also necessary to design public policies to reduce the vulnerability of children and their families. Government actions have proven important in reducing the repercussions of the pandemic on the living conditions of these families; however, they need to be continued to favor the reduction of the vulnerabilities to which they are exposed on a daily basis.

Finally, it is possible to conclude that the data analyzed in light of the theoretical framework in this study made it possible to understand the management experiences experienced by CCC families in the context of the COVID-19 pandemic. Thus, it is recommended that the FMSF be used or that its structuring concepts be used to guide the care provided to children and their families due to the potential to contribute to the understanding of their realities and promote care that meets their needs.

## Data Availability

The research data are available within the article.
