# Supplementary material for: Family management of care for children with chronic conditions in the context of the COVID-19 pandemic
Source: Rev Bras Enferm. 2025 Oct 3;78(Suppl 2):e20240550. doi: 10.1590/0034-7167-2024-0550 (PMC12500071; doi:10.1590/0034-7167-2024-0550)
Supplement: Translation

# Translation: Manejo familiar do cuidado de crianças com condições crônicas no contexto da pandemia de COVID-19

**DOI:** 10.1590/0034-7167-2024-0550pt

**Authors:** Nayara Luiza Henriques, Maísa Mara Lopes Macêdo, Melissa Joice de Abreu Felizardo, Elaine Cristina Rodrigues Gesteira, Elysângela Dittz Duarte

**Affiliations:** I Universidade Federal de Minas Gerais. Belo Horizonte, Minas Gerais, Brasil; II Universidade Estadual de Campinas. Campinas, São Paulo, Brasil

**Keywords:** Doença Crônica, Cuidado da Criança, Família, COVID-19, Pesquisa Qualitativa.

## Abstract

**Objetivos:**

conhecer a experiência de manejo familiar no cuidado de crianças com condições crônicas no contexto da pandemia de COVID-19.

**Métodos:**

estudo qualitativo, baseado no *Family Management Style Framework*, realizado com 24 famílias de crianças com condições crônicas. Os dados foram coletados por entrevista semiestruturada e submetidos à análise temática.

**Resultados:**

anteriormente à pandemia, as famílias seguiam as suas rotinas, adaptando-se às demandas de cuidados. Com a pandemia, novos desafios na dinâmica familiar foram impostos, influenciando os comportamentos de manejo. O enfrentamento e a resiliência foram determinantes para manter o foco no bem-estar de seus filhos no contexto da crise.

**Considerações Finais:**

o conhecimento sobre o manejo familiar do cuidado de crianças com condições crônicas no contexto da pandemia permitiu reconhecer os esforços das famílias para o cuidado em situação adversa. Novas habilidades e competências foram adquiridas, mas houve um impacto para a continuidade do cuidado.

## INTRODUÇÃO

O crescimento do número de crianças que convivem com uma condição crônica é uma realidade identificada nos últimos anos^(1)^. Isso pode ser atribuído a fatores como a crescente especialização do cuidado infantil e os avanços tecnológicos, que permitem diagnósticos e tratamentos mais apurados^(2)^. Quando a saúde da criança está estável, o domicílio é o principal cenário de cuidado, e a família assume a responsabilidade por essa tarefa^(3,4)^.

Ao longo da vida, processos de adaptações e readaptações serão vivenciados pelas famílias de crianças com condições crônicas (CCCs)^(5)^. Isso ocorre porque a condição de saúde da criança pode variar, assim como as finanças e a rede de apoio da família, de forma que a organização familiar para o cuidado sofrerá mudanças ao longo do tempo e conforme o contexto no qual a CCC e seus cuidadores estão inseridos^(3)^. O contexto, portanto, é entendido como o ambiente no qual a criança e família vivem^(6)^.

As enfermeiras Kathleen Knafl e Janet Deatrick, autoras do *Family Management Style Framework* (FMSF)^(7)^, propõem a definição de contexto como sendo uma dimensão para o cuidado familiar, uma vez que ele poderá interferir na condição de vida da família e na maneira como ela percebe e gerencia o cuidado^(7)^.

Uma mudança significativa no contexto foi vivenciada globalmente com a pandemia devido à doença do coronavírus 2019 (COVID-19), ocasionada pelo vírus SARS-CoV-2^(8)^. Embora a literatura indique que o público infantil tenha sido menos afetado pela forma grave e sintomática da COVID-19, as CCCs possuem risco aumentado para complicações mais graves e hospitalizações devido à infecção^(9)^.

No contexto das famílias de CCCs, as metas de cumprir todas as determinações sanitárias e conseguir dar continuidade ao cuidado foram consideradas um desafio^(10)^. As demandas adicionais decorrentes da pandemia, como cuidados relacionados à higiene, uso de máscara e necessidade de distanciamento social, geraram maior sobrecarga para os cuidadores e aumento dos níveis de estresse^(11,12)^. Além dessas demandas, a família precisou lidar com a redução da rede de apoio, do convívio familiar e dos atendimentos por profissionais de saúde^(13)^.

Face a este contexto, considerou-se a necessidade de investigar como as famílias se organizaram para garantir o cuidado da CCC no contexto da pandemia de COVID-19. Para tanto, utilizou-se o FMSF como subsídio para essa investigação. Acredita-se que conhecer a experiência de manejo do cuidado pelas famílias de CCCs durante a pandemia de COVID-19 permite identificar as potencialidades da família como núcleo social de cuidado, sua capacidade de resistir e se adaptar, ou não, a esse tipo de mudança contextual, e os aspectos que podem ter sido fragilizados durante a pandemia, colocando em risco o funcionamento familiar.

Sendo assim, considera-se que as modificações produzidas no contexto das famílias, acarretadas pela pandemia de COVID-19, acentuaram os esforços familiares empreendidos para o atendimento das demandas de cuidado da CCC. A partir desta assertiva, definiu-se a pergunta de pesquisa: como foi o manejo familiar do cuidado de CCCs durante a pandemia de COVID-19?

## OBJETIVOS

Conhecer a experiência de manejo familiar no cuidado de crianças com condições crônicas no contexto da pandemia de COVID-19 à luz do referencial teórico do *Family Management Style Framework*.

## MÉTODOS

### Aspectos éticos

O projeto foi submetido e aprovado pelo Comitê de Ética em Pesquisa da instituição proponente. A fim de assegurar a adequação do estudo às normas éticas de pesquisas que envolvem seres humanos, foram respeitadas as Resolução nº 466/2012 do Conselho Nacional de Saúde (CNS) e as diretrizes do Ofício Circular nº 2/2021^(14)^ da Comissão Nacional de Ética em Pesquisa. O Termo de Consentimento Livre e Esclarecido (TCLE) foi obtido de todos os indivíduos envolvidos no estudo por meio de expressão oral, visto que as entrevistas foram realizadas por telefone devido ao cenário de distanciamento social. Assim, foi realizada a leitura do TCLE, e o consentimento de participação no estudo foi gravado e arquivado em mídia segura, conforme previsto nas Resoluções nº 466/2012 e nº 580/2018 do CNS.

### Referencial teórico-metodológico

Adotou-se como referencial teórico para este estudo o FMSF^(7)^, visto que ele possibilita uma análise das respostas das famílias perante as necessidades vivenciadas pela existência de uma condição crônica na infância, considerando a influência do contexto na vida familiar através dos seguintes componentes: definição da situação (significados atribuídos pela família na experiência da doença, identificados pelas dimensões: identidade da criança, visão da condição, mentalidade de manejo e mutualidade parental); comportamentos de manejo (comportamentos adquiridos pelos membros familiares em diferentes situações expostas pela condição, identificados pela filosofia parental e abordagem de manejo); e consequências percebidas (resultados atuais e esperados pela família sobre o manejo da condição, identificados pelo foco familiar e expectativas futuras)^(7)^.

### Tipo de estudo

Trata-se de estudo qualitativo, construído com base nos critérios do *Consolidated Criteria for Reporting Qualitative Research* ^(15)^.

### Procedimentos metodológicos

A pesquisa foi realizada com 24 famílias de CCCs. Inicialmente, um representante de cada família foi entrevistado, sendo 22 mães, um pai e uma tia, considerados os cuidadores principais da criança, ou seja, aqueles que assumem mais intensamente a responsabilidade pelo cuidado dela. Para contemplar diferentes perspectivas da família, foi solicitada a esses cuidadores a indicação de outro membro familiar que também tinha envolvimento no cuidado da criança. Em cinco famílias, não havia outro cuidador; três não responderam à solicitação; e 16 cuidadores indicaram outro familiar. Dessas 16 famílias, em nove, foi possível entrevistar outro familiar, sendo seis pais, uma mãe, um tio e uma avó. Logo, um total de 33 entrevistas foram realizadas. Cabe ressaltar que, embora a pesquisa não tenha sido realizada com todos os membros das famílias, a produção das informações se referiu à família como um todo.

Os critérios de inclusão para participação na pesquisa foram ser familiar da CCC e estar envolvido com o seu cuidado, com mais de 18 anos, capaz de ouvir, compreender e verbalizar as respostas à pesquisadora, possuindo viabilidade para contato telefônico. O critério de exclusão foi a não obtenção de resposta do familiar após três tentativas de contato da pesquisadora. Adotou-se o critério de saturação temática para a interrupção da coleta de dados^(16)^.

### Cenário do estudo

As famílias participantes foram identificadas por meio de registros de internação hospitalar de crianças que receberam alta da Unidade de Terapia Intensiva Neonatal (UTIN), entre dezembro de 2016 e dezembro de 2017, de dois hospitais de referência em saúde materno-infantil na capital de Belo Horizonte, Minas Gerais, Brasil. O período foi definido para possibilitar a identificação de crianças entre 2 e 4 anos, faixa etária em que as alterações no desenvolvimento neuropsicomotor são mais notáveis pelas famílias^(17)^.

### Fonte de dados

A identificação das crianças ocorreu entre outubro de 2019 e maio de 2020, para um estudo primário, realizado no contexto anterior à pandemia de COVID-19^(18)^. Foram localizadas 1.115 famílias. Posteriormente, realizou-se contato telefônico, com todas essas famílias, para aplicação do Questionário para Identificação de Crianças com Condições Crônicas - Revisado (QuICCC-R)^(19)^. No total de famílias, não foi possível entrar em contato com 829, devido ao número de telefone ser inexistente ou não pertencer mais ao familiar. Foram contatadas 286 famílias, sendo que, dessas, cinco crianças haviam falecido; 219 não possuíam condições crônicas de acordo com o QuICCC-R^(19)^; e nove famílias se recusaram a participar. Desse modo, 53 famílias de CCCs participaram do estudo primário^(18)^.

Para esta pesquisa, foram feitas tentativas de contato com as mesmas 53 famílias participantes do estudo primário^(17)^. Dessas, nove famílias não puderam ser contatadas, pois o número telefônico não era válido; 13 não responderam após três tentativas de ligações; e quatro não desejaram participar. Assim, a coleta de dados foi feita com 27 famílias, contudo três participaram de teste piloto e não fizeram parte da análise dos dados, restando, assim, 24 famílias.

### Coleta e organização dos dados

A coleta de dados aconteceu entre janeiro e maio de 2021, por meio de entrevista semiestruturada. Devido às medidas de prevenção de infecção pela COVID-19, optou-se por produzir os dados por meio de contato telefônico, o que assegurou o acesso e alcance das famílias no contexto de isolamento social. Para assegurar a efetividade da entrevista, os participantes foram contatados, inicialmente, pela pesquisadora do estudo primário com quem já possuíam familiaridade. Na ocasião, ela apresentou este estudo e as pesquisadoras envolvidas nele, facilitando o contato. Reconhecendo-se a possibilidade de limitações da entrevista por telefone, como distrações do participante, problemas técnicos e de conexão, falta de comunicação não verbal e menor vínculo entre pesquisador e entrevistado, foram adotadas estratégias como agendar previamente a entrevista para minimizar distrações, manter contato com os entrevistados via mensagens de texto pelo *WhatsApp^®^* para fortalecer o vínculo e construir um roteiro de entrevista com perguntas para favorecer a adequada condução da entrevista.

O roteiro de entrevista foi elaborado segundo os componentes do FMSF^(7)^, com perguntas sobre o cuidado diário da criança durante a pandemia, com foco nas mudanças ocorridas nas demandas de cuidado, dinâmica familiar, mutualidade familiar, acesso a serviços de saúde e perspectiva sobre o futuro da criança. As entrevistas foram realizadas por uma das autoras deste estudo, após contato telefônico prévio com os participantes para agendamento de dia e horário de sua preferência. A pesquisadora é enfermeira, mestre e doutoranda em enfermagem, com experiência na condução de entrevistas qualitativas, e embora atuasse no atendimento de crianças em um dos hospitais nos quais os participantes foram identificados, não teve contato prévio com eles.

A entrevista foi realizada, individualmente, com cada membro da família participante. Atentou-se para a permanência de um ambiente silencioso e com boa cobertura de sinal telefônico. O tempo médio de duração das entrevistas foi de 15 minutos e 34 segundos. Todas as entrevistas foram transcritas na íntegra. Para validação de cada transcrição, realizou-se a leitura do texto com a escuta do áudio.

### Análise dos dados

Os dados foram submetidos à análise temática^(20)^, com abordagem dedutiva guiada pelo FMSF. Os documentos das entrevistas transcritas foram exportados para o *software MAXQDA^®^* ^(21)^ para a codificação, exploração e gerenciamento dos dados qualitativos.Para a construção da análise temática, utilizaram-se as seis etapas propostas por Braun e Clarke^(20)^. Elencaram-se, inicialmente, temas correspondentes aos componentes e dimensões do FMSF^(7)^. Esses temas foram aplicados às transcrições da entrevista e, posteriormente, buscou-se a identificação de aspectos que sinalizavam padrões na experiência das famílias. Para a validação da atribuição dos temas, as cinco primeiras entrevistas foram codificadas de forma independente e às cegas por duas pesquisadoras, sendo a primeira e segunda autora deste estudo. Nesta etapa, obteve-se índice Kappa de 0,95, que representa alto nível de concordância intercodificador^(22)^. As divergências encontradas foram discutidas com uma terceira pesquisadora para consenso. As demais entrevistas foram codificadas pela segunda autora deste artigo.

## RESULTADOS

### Caracterização dos participantes

Participaram 24 famílias de CCCs. Considera-se que, sendo o cuidador principal a pessoa quem assume mais intensamente o cuidado da criança, ele pode oferecer informações sobre a perspectiva da família. Sendo assim, as características sociodemográficas que serão apresentadas são referentes aos cuidadores principais. A maioria (n=22) das entrevistas foi respondida por mães, das quais 41% eram donas de casa. Entre os cuidadores principais (n=24), a faixa etária predominante foi de 31 a 40 anos (n=17). A maioria (n=8) concluiu o ensino médio, seguida pelo ensino superior. Em relação à etnia, quatro eram brancos, quatro, negros, e 16, de outras etnias. A maioria (n=21) tinha alguma religião e vivia com um companheiro (n=18). A renda familiar se baseava no salário mínimo (R$1.100,00), com 25% recebendo menos que isso. Dezessete famílias recebiam algum tipo de auxílio financeiro.

Em relação às 25 crianças participantes, suas idades variaram entre 3 e 4 anos, com idade gestacional média de 31,8 semanas, sendo nove prematuras. A maioria era do sexo masculino (n=17), e 12 frequentavam a escola. Todas recebiam acompanhamento especializado em saúde, com a maioria (n=13) necessitando de múltiplas especialidades. Os diagnósticos incluíam condições neurológicas e/ou neuromusculares (n=13), condições cardiovasculares (n=3), condições respiratórias (n=1) condições gastrointestinais e endócrinas (n=1), condições renais (n=1), condições ósseas e/ou das articulações (n=4), e anomalias congênitas e defeitos genéticos (n=2). Crianças com hidrocefalia utilizavam derivação ventrículo-peritoneal.

### Family Management Style Framework

A análise dos dados permitiu identificar, a partir dos componentes do FMSF, elementos que compuseram a experiência de cuidado das crianças pelas famílias durante a pandemia de COVID-19. A Figura 1 possibilita a visualização de aspectos desta experiência, bem como das mudanças vivenciadas nesse novo contexto de cuidado.

**Figura 1:** *Family Management Style Framework* de crianças com condições crônicas no contexto da pandemia de COVID-19, Belo Horizonte, Minas Gerais, Brasil, 2024

*Fonte: adaptado de Knafl, Deatrick e Havill (2012).*

### Definição da situação

Esse componente destinou-se a identificar como as famílias vivenciaram o cuidado das CCCs durante a pandemia de COVID-19. Segundo o FMSF, a definição da situação se relaciona ao significado que os membros da família conferem aos elementos importantes da situação vivenciada, que neste estudo é especificamente o cuidado de uma CCC no contexto da pandemia. Quatro dimensões conformam este componente: identidade da criança; visão da doença; mentalidade de manejo; e mutualidade familiar.

A identidade das crianças foi expressa por meio das fragilidades identificadas pelos familiares considerando as condições de saúde pré-existentes e a sua contribuição para maior suscetibilidade à COVID-19. A visão da doença manifestada pelas famílias refletiu um prognóstico negativo para as crianças, devido à incerteza do fim da pandemia, informações negativas na mídia sobre infectados, desconhecimento sobre as consequências da doença e incerteza sobre a vacinação. A mentalidade de manejo das famílias durante a pandemia evidenciou dificuldades decorrentes da interrupção de atendimentos, fechamento de escolas e aumento do estresse das crianças por permaneceram maior tempo no domicílio. Apesar dos desafios impostos pela pandemia, a mutualidade familiar se destacou pela oportunidade de união, companheirismo e participação no cuidado entre membros das famílias. As informações estão exemplificadas no Quadro 1.

**Quadro 1:** Definição da situação das famílias para manejar as crianças com condições crônicas durante a pandemia de COVID-19, Belo Horizonte, Minas Gerais, Brasil, 2024

Categoria: definição da situação		
**Dimensões**	**Experiência de manejo familiar**	**Exemplos de enunciados**
**Identidade da criança:** refere-se ao quanto o foco da família se concentra nas capacidades ou nas fragilidades da criança.	Fragilidades determinadas pela condição crônica, levando a maior susceptibilidade para a infecção por COVID-19.	[...] *eu tenho medo dele pegar COVID, e como ele tem problema respiratório, dele ter uma dificuldade muito extrema para respirar* [...]. (M43) *Mudou muitas coisas porque a gente fica com medo, sabe? Ela é muito frágil. Se ela pegar esse trem, eu acho que vai ser terrível, viu?* (P22)
**Visão da doença:** relacionada às crenças da família quanto à causa, gravidade, previsibilidade e curso da condição de saúde da criança.	Crenças das famílias de que as condições crônicas já existentes são graves e que podem ser potencializadas caso as crianças sejam contaminadas, o que causa angústia e medo.	*Ela tem displasia pulmonar. Então, ela ter essas lesões do pulmão, que me deixa receosa com a COVID, por ser um vírus respiratório.* (M5) *Se ele pegar o vírus, pode piorar. A preocupação maior está relacionada a isso, né? Eles falaram que não vai ter vacina para ele e nem nada, que não vão dar a vacina às crianças. Eu achei, assim, um absurdo, né? Ele precisa da vacina.* (M24)
**Mentalidade de manejo:** facilidades e/ou dificuldades em realizar o regime terapêutico da criança, bem como sua capacidade para manejá-lo de maneira eficaz.	As facilidades adquiridas ao longo da experiência de manejo antes da pandemia foram impactadas pela interrupção dos serviços assistenciais, tornando-se difícil o seguimento do regime terapêutico.	*Como se diz, nesse momento da pandemia, a gente teve que parar com a aula de natação. O ambulatório do Hospital das Clínicas, a gente não teve consulta aqui na APAE. O médico também parou, então, assim, o cuidado ficou difícil* [...]. (M50) *Tá sendo, assim, uma correria, né? Mais estressante, porque não tem escola, né? Então, eles ficam em casa mais ansiosos.* (M20)
	Necessidade de manutenção das terapias no domicílio pelos próprios cuidadores.	*Uai, tá sendo assim, mais delicado, porque ela não está tendo fisioterapia, não é? Não está tendo esses atendimentos presenciais. Aí, no caso, está tendo fisioterapia online, né? No caso, eu que estou tendo que fazer fisioterapia.* (M25)
**Mutualidade familiar:** corresponde às percepções, compartilhadas ou não, sobre a criança, a condição e a abordagem de manejo.	Os membros familiares já tinham adquirido maior envolvimento nos cuidados das crianças, tornando-se mais unidos, porém, com a pandemia, essa vivência se intensificou.	*Ele também me ajuda muito* [se refere ao esposo]. *Só na sonda que não, porque, tipo assim, ele tem muita insegurança em questão da sonda.* (M25) *Na verdade, até antes da pandemia, a gente se uniu mais por causa da C5. Nós nos dedicamos muito a ela, muito mesmo.* (P5)

### Comportamentos das famílias para manejar o cuidado das crianças com condições crônicas durante a pandemia de COVID-19

Os comportamentos de manejo se referem aos esforços da família para cuidar e se adaptar às demandas decorrentes da condição crônica. Duas dimensões ajudam a compreender este componente: filosofia dos cuidadores; e abordagem de manejo.

A filosofia dos cuidadores abrange suas crenças, valores e prioridades, influenciando diretamente a abordagem de manejo. Neste estudo, identificou-se que a crença na suscetibilidade da criança à COVID-19 foi determinante para a intensificação dos cuidados para prevenção da infecção e manutenção da sua saúde (abordagem de manejo), como evitar sair de casa, limitar visitas e intensificar medidas de higiene. A crença (filosofia dos cuidadores) de que a falta de estímulos profissionais e socialização pode levar a atrasos no desenvolvimento da criança fez com que as famílias incorporassem atividades de estímulo no domicílio (abordagem de manejo). As informações sobre este componente se encontram exemplificadas no Quadro 2.

**Quadro 2:** Comportamentos das famílias para manejar o cuidado das crianças com condições crônicas durante a pandemia de COVID-19, Belo Horizonte, Minas Gerais, Brasil, 2024

Componente: comportamento de manejo		
**Dimensões**	**Experiência de manejo familiar**	**Exemplos de enunciados**
**Filosofia dos cuidadores:** objetivos, prioridades, valores e crenças dos cuidadores que orientam a abordagem geral e estratégias específicas para o manejo do cuidado da criança.	Crença de que a criança é mais vulnerável e suscetível à COVID-19.	*Bem, a gente redobrou os cuidados por ela ter essa parte da gastrostomia. A gente preocupa muito.* (P2) *Então, mais cuidado mesmo, agora é dobrado. O cuidado que eu tinha com ele mais era a alimentação. Agora, né, eu tenho que evitar sair em público, usar álcool em gel, limpeza ao máximo.* (M17)
	Crença de que a falta de estímulos de profissionais especializados e impossibilidade de socialização podem levar a atrasos no desenvolvimento.	*Ah, de uma forma ou não, o desenvolvimento prejudica, né, porque parou tudo, parou escola, parou fonoaudiólogo, fisioterapia, então, querendo ou não, tá prejudicando tudo, porque não tem todos os recursos.* (M16) *Minha filha vai ser prejudicada em todos os sentidos. Ela precisa reforçar os passos, é terapia ocupacional, é fisioterapia, e até hoje nada. Ela precisa!* (M5)
**Abordagem de manejo:** desenvolvimento de estratégias e rotinas para o manejo da condição da criança.	Adoção de comportamentos para o cumprimento do distanciamento social, intensificação de medidas para prevenção e contenção da COVID-19.	*A gente evita ficar recebendo muita gente em casa por causa da C41. A gente não sai, sai só quando precisa.* (M41) *A gente já tem medo por conta da imunidade dela, que já é baixa, né? E se eu precisar de sair eu deixo ela em casa.* (M51) *Caso tenha que sair, precisa das máscaras, precisa ter cuidado com álcool em gel, não pode ficar deixando ninguém tocar nele.* (M24)
	Adoção de comportamentos para a manutenção das atividades de estímulo da criança no domicílio.	*A fisioterapia, assim, eu faço com ela aqui em casa. A gente brinca muito, eu mexo com ela, então ela acaba fazendo fisioterapia.* (M22) *Ele tinha feito oito sessões. A fisioterapeuta parou de atender, aí ela pediu pra continuar fazendo em casa.* (M53)

### Consequências percebidas pelos familiares quanto a cuidar de uma criança com condições crônicas no contexto da pandemia de COVID-19

Este componente diz respeito à avaliação da família sobre o impacto da condição crônica e dos cuidados decorrentes no funcionamento familiar e nas expectativas futuras. Neste estudo, esta avaliação considerou as mudanças na dinâmica familiar devido à pandemia de COVID-19. Segundo o FMSF, o componente tem duas dimensões: foco da família; e expectativas futuras.

No que se refere ao foco da família, que envolve a satisfação com a incorporação do manejo da condição da criança na vida familiar, os cuidadores relataram um equilíbrio na rotina de cuidados, apesar do aumento das responsabilidades com as medidas de contenção da COVID-19. Eles se concentraram em proteger, preservar a saúde e promover o bem-estar da CCC, mantendo as atividades habituais de cuidado e compensando aquelas afetadas pela pandemia como estímulo ao desenvolvimento e reabilitação. A maioria dos cuidadores expressou satisfação com a nova rotina, atribuindo-a aos esforços para garantir a qualidade do cuidado. Contudo, alguns manifestaram insatisfação, sentindo-se inadequadamente capacitados em comparação aos profissionais de saúde e educação.

Quanto às expectativas futuras, todos os cuidadores foram indagados sobre o futuro da criança e da família no contexto da pandemia. Apenas 12 responderam, enquanto os demais foram evasivos, possivelmente devido à incerteza da situação. As expectativas futuras manifestadas incluíam o desejo de que a criança não contraísse o vírus, a esperança pelo fim da pandemia e o início da vacinação. As informações sobre este componente estão exemplificadas no Quadro 3.

**Quadro 3:** Consequências percebidas pelos familiares quanto a cuidar de crianças com condições crônicas no contexto da pandemia de COVID-19, Belo Horizonte, Minas Gerais, Brasil, 2024

Componente: consequências percebidas		
**Dimensões**	**Experiência de manejo familiar**	**Exemplos de enunciados**
**Foco na família:** avaliação e satisfação da família com a forma como o manejo da condição da criança foram incorporadas à rotina familiar.	Avaliação de que o cuidado da criança foi sustentado mediante a construção de uma rotina familiar ajustada.	*A M18 é o pilar aqui dentro de casa. Eu lá fora ralando e ela aqui dentro com ele, dando aula, educando, cuidando.* (P18) *Eu praticamente fico por conta dela, né? O tempinho que ela não tem nada, eu vou ali lavar uma roupa né, ou arrumar a casa.* [...] *meu dia e minha noite. Eu vivo para ela.* (M8)
	Avaliação de satisfação pelo cuidado ofertado.	*A gente tenta oferecer 100% no cuidado. Claro que a gente não consegue, mas 90%, o nosso melhor, a gente consegue.* (M18) *Ninguém consegue ser 100%, mas tentamos ser o máximo possível observadores, atenciosos e responsáveis pela situação, tomando os cuidados.* (P2)
	Insatisfação pelo cuidado ofertado devido à crença de não conseguir realizar bem algum cuidado.	[...] *parou tudo, parou escola, parou fonoaudiólogo, fisioterapia, então, querendo ou não, tá prejudicando tudo, né, porque não tem todos os recursos. Aqui em casa, eu converso, tento, estímulo ela bastante, brinco, mas não é a mesma coisa que um profissional fazer com ela, sabe, estar com ela.* (M16)
**Expectativas futuras:** avaliação da família sobre as implicações da condição para o futuro da criança e da família.	Expectativa da não contaminação da criança.	*Eu espero que a L. não pegue, porque eu tenho muito medo. Ela tem o coraçãozinho furado, tem problema pulmonar, um monte de coisinha, sabe?* (P8)
	Esperança pela chegada da vacina contra a COVID-19.	*Eu estou torcendo por essa vacina, porque, nossa, para deixar essa preocupação para trás* [...]. (M3) *Olha, agora, com a vacina chegando, né, estamos um pouco esperançosos, né, mas vou te contar que é muito estresse* [...]. (M17)
	Esperança pelo fim da pandemia.	[...] *torcer para a pandemia acabar logo, todo mundo tomar a vacina, né, ele ir para a escolinha!* (A24)

## DISCUSSÃO

A pandemia de COVID-19 desencadeou mudanças substanciais na rotina das famílias que cuidam de CCCs, influenciando o manejo para o cuidado diário e imputando novas necessidades. Conhecer a maneira como as famílias incorporaram o cuidado da CCC à vida familiar cotidiana, bem como as adversidades para cuidar em meio a uma crise sanitária, explicitou a necessidade de oferecer apoio a essas famílias, partindo do reconhecimento de como elas percebem e avaliam a CCC e o cuidado ofertado, além dos recursos que utilizam como fonte de apoio e orientação.

Observa-se, neste estudo, que as famílias que enfrentaram desafios mais intensos relacionados ao cuidado da CCC na pandemia foram aquelas com menor renda familiar mensal e que possuíam crianças com condições neurológicas complexas, pois essas famílias já enfrentavam dificuldades financeiras antes do advento da pandemia, pois precisavam se dedicar integralmente ao cuidado da criança. Com a pandemia, precisaram incorporar novos cuidados à rotina, além de vivenciar a perda da rede de apoio, o que agravou a situação de vulnerabilidade social, financeira e de sobrecarga emocional. Sabe-se que famílias que têm CCCs possuem uma condição financeira mais vulnerável, decorrente, entre outros, da interrupção do trabalho remunerado para dedicarem ao cuidado, além de gastos com a saúde. Esses acontecimentos podem acentuar a redução da renda familiar, comprometendo o manejo e a adaptação da família às situações adversas^(23)^.

Como estratégia para mitigar a crise econômica resultante da pandemia, visando garantir renda mínima e segurança alimentar, o governo brasileiro ofereceu o auxílio emergencial. Esse incremento financeiro foi destinado às famílias em situação vulnerabilidade econômica^(24)^. Neste estudo, a maior parte das famílias recebeu esse auxílio. Embora essa ação governamental pareça ter contribuído para manter uma estabilidade econômica das famílias de CCCs em situação de vulnerabilidade, esse grupo precisa ser alvo de estratégias efetivas e continuadas de seguridade social, não se limitando apenas aos períodos de crises. É importante ressaltar que famílias que referiram melhor poder aquisitivo nesta pesquisa também enfrentaram mudanças na rotina de cuidados, uma vez que, com as circunstâncias da pandemia, não foi possível manter a normalidade da rotina.

A análise dos dados baseada no FMSF evidenciou a existência de uma interconexão das dimensões do referencial teórico, o que foi essencial para compreender as mudanças ocorridas na rotina de cuidado das famílias durante a pandemia. Os achados deste estudo indicam que a pandemia influenciou os cuidadores a enfatizarem as fragilidades de suas crianças em detrimentos das suas potencialidades. Ao atribuírem esta identidade à criança, também fica reforçada a sua crença de que elas são mais suscetíveis à COVID-19 e às suas repercussões, além da visão de que as condições crônicas já existentes podem ser potencializadas caso as crianças sejam contaminadas. Isso conduziu as famílias a experimentarem sentimentos de medo e preocupação, e a adotarem comportamentos de manejo visando à proteção da criança contra a infecção pelo SARS-CoV-2, como uso de álcool em gel, máscara e distanciamento social, com a expectativa futura de que a criança não se contaminasse.

As crenças que definem a visão da família sobre a condição da criança podem ser modificadas a depender do contexto no qual a família se insere^(7)^. Considerando o período de coleta dos dados, acredita-se que a incerteza do fim da pandemia, as más notícias propagadas pela mídia, a insegurança quanto à infecção e suas consequências, e a incerteza de quando a vacina seria uma realidade podem ter contribuído para a crença de um mau prognóstico das CCCs. Ao contrário dos adultos, a maioria das crianças infectadas apresentou um curso mais brando da doença. No entanto, a maioria das mortes, hospitalizações e consequências críticas relatadas em crianças ocorreu naquelas com comorbidades prévias^(9)^, dados que justificam maior preocupação dos cuidadores com suas crianças, assim como a adoção de cuidados para prevenção da COVID-19.

A interrupção dos atendimentos em saúde e o fechamento das escolas, dificuldades identificadas pelos cuidadores, foram atrelados à crença na possibilidade de prejuízos no desenvolvimento da criança. Como abordagem de manejo, as famílias se esforçaram para realizar atividades de estimulação em casa e permaneceram mais unidas, como evidenciado na dimensão mutualidade familiar. Sendo assim, os resultados permitem reconhecer que já existia conhecimento e habilidade dos cuidadores, anteriores à pandemia, resultantes de um aprendizado contínuo e de constantes adaptações sobre as necessidades de sua criança e o cuidado, dando suporte às ações quando o acesso aos serviços de saúde e educação foi perdido.

Estudos indicam que a pandemia de COVID-19 produziu impactos significativos para o desenvolvimento infantil, sendo as crianças em idade pré-escolar e escolar as mais afetadas. Aumento da exposição a telas, redução das brincadeiras ao livre e das atividades educacionais, aumento da insegurança alimentar, e redução do contato social com a família e amigos foram identificados como causadores de prejuízos socioemocionais, linguísticos, de crescimento e desenvolvimento referentes à saúde mental^(25,26)^. Este contexto indica a necessidade de que as famílias sejam apoiadas para o manejo das crianças em situações que modificam os seus contextos de vida, reduzindo os impactos sobre o desenvolvimento infantil. A exemplo, tem-se a implementação de ações como teleconsultas, suporte educacional remoto, programas de capacitação de cuidadores e criação de materiais, e tecnologias acessíveis que atuem favorecendo o desenvolvimento das crianças no domicílio.

Os participantes deste estudo relataram ainda que os membros da família permaneceram por mais tempo no domicílio durante a pandemia de COVID-19, fato reportado como dificuldade, mas que também proporcionou mais convivência familiar e participação no cuidado da CCC. A percepção dos participantes sobre as “consequências percebidas” evidencia satisfação pelo esforço conjunto na adoção de medidas protetivas contra a COVID-19. Para algumas famílias, esta percepção é potencializada pelo reconhecimento da mutualidade familiar que, além do compartilhamento do cuidado, teve acentuada a união e companheirismo entre os membros da família. Cabe considerar que a mutualidade familiar é resultado de um processo contínuo de construção dentro da família que pode ter sido fortalecido durante o período pandêmico, mas não se limita a ele.

A maior parte dos entrevistados possuía ensino médio ou superior, aspecto que pode ter contribuído para o cuidado da criança, uma vez que existe uma relação positiva entre os anos de estudo e a habilidade de manejar o cuidado da criança^(23)^. A competência dos cuidadores para o cuidado foi uma componente central verificada no comportamento familiar na dimensão “abordagem de manejo”. Embora esteja evidenciada a relevância desta competência para redução de danos e potência da família enquanto sustentáculo do cuidado de CCC, ela merece atenção, especialmente a médio e longo prazo, em situações sanitárias semelhantes. Um dos aspectos a serem considerados é o preparo dos cuidadores para um atendimento a necessidades já conhecidas. As mudanças no desenvolvimento e na saúde das crianças exigem ações que os cuidadores podem não ter experimentado anteriormente.

Sendo assim, embora as intervenções realizadas no domicílio tenham desempenhado um papel importante na reabilitação da criança em um contexto adverso, essa estratégia pode não se sustentar com o passar do tempo. Os cuidadores podem não ter sido capacitados para atuar de maneira adequada para alcançar os resultados funcionais desejados, justificando a insatisfação quanto ao cuidado referida por alguns deles. Além disso, ainda que capacitados, o atendimento da criança por um profissional qualificado não é dispensável, visto que é realizado, de forma individualizada, conforme o desenvolvimento de cada criança, sendo importante que a família reconheça esse aspecto.

A interrupção ou modificação de atendimentos destinados à reabilitação da criança tem o potencial de trazer prejuízos à sua capacidade funcional^(27)^. Neste estudo, fica evidente que as famílias reconhecem esse impacto, justificando a preocupação com a manutenção e garantia das atividades de estímulo. Recursos que facilitem o compartilhamento de cuidados a distância devem ser mais utilizados, como os serviços de telessaúde e uso de plataformas com videochamadas, visando à prevenção dos efeitos negativos de futuras emergências de saúde que demandem distanciamento social.

Estudos apontam que a perda do apoio oferecido pela escola foi um dos maiores desafios para as famílias de CCCs^(28,29)^. Nesta investigação, este desafio foi determinante para a adoção de comportamentos enquanto abordagem de manejo, como maior dedicação de tempo às atividades escolares no domicílio, visando minimizar os impactos da falta do ensino e da socialização das crianças nas escolas. Fatores como dificuldades de aprendizagem, crianças de diferentes idades em casa e a necessidade de trabalhar podem ter exacerbado os desafios^(26)^.

A impossibilidade de socialização da criança com seus pares, ocasionada pelo fechamento das escolas e distanciamento social, foi outro motivo de preocupação para alguns cuidadores. O confinamento produzido pela pandemia trouxe limitações à vida das crianças, restringindo seu ambiente ao domicílio e à interação com a família. De fato, a privação da interação social das crianças pode produzir perdas no seu aprendizado e desenvolvimento. Além disso, neste estudo, os cuidadores perceberam que a falta de interação com outras crianças pode ter sido motivo de irritabilidade, medo e mudanças de comportamento em suas crianças, achados consonantes com estudo com crianças e adolescentes com doença falciforme^(30)^.

### Limitações do estudo

É reconhecida como limitação do estudo a população selecionada, considerada pouco diversificada. Investigações com famílias de CCCs específicas e com demandas de cuidado mais complexas podem produzir resultados distintos dos que foram encontrados neste estudo.

### Contribuições para a área da enfermagem

Este estudo contribui para a enfermagem pois utiliza o FMSF como um modelo teórico que pode direcionar as práticas do enfermeiro, proporcionando reflexões críticas sobre: a dinâmica de cada família, incluindo a visão dela sobre a criança e sua condição de saúde; os esforços necessários para estabelecer uma rotina e atender às demandas da CCC; o impacto da condição de saúde da CCC para a família e para o futuro; e a influência do contexto no qual a família se insere.

## CONSIDERAÇÕES FINAIS

A pandemia levou a mudanças na vida cotidiana das famílias de CCCs, produzindo desafios únicos a serem enfrentados. A mudança de rotina foi necessária, sobretudo devido à incorporação de cuidados para a prevenção e contenção da COVID-19, como medidas de higiene, uso de máscaras e distanciamento social. Além disso, as famílias vivenciaram a interrupção dos atendimentos de saúde e o fechamento das escolas, que foram vistos como uma dificuldade, devido ao potencial de trazer prejuízos ao desenvolvimento da criança.

A mudança substancial e rápida no modo de oferta e acesso ao apoio por parte dos profissionais de saúde e educação aumentou a sobrecarga dos cuidadores, que passaram a se dedicar ao ensino de atividades escolares e reabilitação no domicílio. Contudo, apesar das dificuldades vivenciadas, as famílias se esforçaram para garantir o cuidado de criança, tornando-se mais unidas diante do maior tempo de permanência no domicílio.

Destaca-se que, apesar de a família ser a protagonista do cuidado no âmbito domiciliar e possuir habilidade e competência para desempenhar essa função, isso não significa que deva assumir essa responsabilidade de maneira exclusiva. A perda do apoio na pandemia deu destaque às capacidades das famílias de CCCs, mas também ressaltou a importância dos profissionais como parceiros desse cuidado. Deste modo, em contextos adversos e de distanciamento social, é preciso pensar em estratégia que fortaleçam essa parceria e assegurem a continuidade do cuidado de maneira estruturada e acessível, utilizando-se, para isto, o conhecimento existente sobre as necessidades das crianças, condições favorecedoras para o desenvolvimento, e mediação da tecnologia tanto para o cuidado direcionado às crianças, a exemplo das teleconsultas, quanto para a educação em saúde de seus cuidadores.

Mesmo reconhecendo a contribuição que as práticas adotadas pelos profissionais podem ter para a qualificação do cuidado em situações de mudanças nos contextos de cuidado, faz-se necessário também que políticas públicas sejam pensadas para reduzir a situação de vulnerabilidade das crianças e suas famílias. As ações governamentais se mostraram importantes para reduzir as repercussões da pandemia na condição de vida dessas famílias; contudo, precisam ser continuadas para favorecer a redução das vulnerabilidades a que estão expostas cotidianamente.

Por fim, é possível concluir que os dados analisados à luz do referencial teórico neste estudo possibilitaram conhecer as experiências de manejo vivenciadas pelas famílias de CCCs no contexto da pandemia de COVID-19. Assim, recomenda-se o uso do FMSF ou que seus conceitos estruturantes sejam utilizados para orientar o cuidado realizado de crianças e suas famílias pelo potencial de contribuir para a apreensão de suas realidades e promover um cuidado que atenda às suas necessidades.

## Data Availability

Os dados da pesquisa estão disponíveis no corpo do artigo.
